# Quenching-based electrochemiluminescence detection of chlorpyrifos using a portable sensor platform

**DOI:** 10.55730/1300-0152.2790

**Published:** 2025-12-09

**Authors:** Emre DOKUZPARMAK

**Affiliations:** Department of Bioengineering, Faculty of Engineering, Ege University, İzmir, Turkiye

**Keywords:** Electrochemiluminescence, quenching-based mechanism, chlorpyrifos, carbon nanotubes, sensor

## Abstract

**Background/aim:**

Chlorpyrifos (CPF) is an organophosphate pesticide extensively used in agriculture, posing significant threats to human health due to its neurotoxic effects. Its presence in food products has raised considerable public health concerns, necessitating the development of rapid, sensitive, and reliable detection methods. This study aims to design and validate an electrochemiluminescence (ECL) sensor platform operating via a quenching-based mechanism to enable ultrasensitive detection of CPF in food matrices.

**Materials and methods:**

The sensing strategy exploits the coreactant mechanism between 2-(dibutylamino)ethanol (DBAE) and [Ru(bpy)_3_]^2+^ (tris(2,2′-bipyridyl)ruthenium(II)), wherein CPF suppresses the ECL emission by interfering with the excited-state electron transfer pathway. The sensing interface was fabricated by integrating carboxyl-functionalized multiwalled carbon nanotubes (MWCNTs) with [Ru(bpy)_3_]^2+^ complexes, which were immobilized on a screen-printed carbon electrode using a Nafion polymer film to enhance stability and conductivity. Analytical parameters, including the limit of detection (LoD), linear range, and reproducibility, were systematically optimized. Real sample applicability was assessed using honey matrices to simulate complex food environments.

**Results:**

The developed sensor achieved a LoD of 0.24 ± 0.05 pM (2.4 × 10^−13^ M) and a linear dynamic range between 0.73 ± 0.13 pM and 100 ± 1.49 pM. In honey samples, recovery rates ranged from 96.26 ± 3.78% to 101.45 ± 4.16%, demonstrating excellent accuracy and minimal interference. The sensor showed good repeatability, with the ECL signal remaining above 80% of its initial value over eight consecutive measurements. Additionally, the relative standard deviation increased slightly from 1.81% to 2.90% over 30 days, indicating high stability of the sensor during the storage period.

**Conclusion:**

This ECL sensor provides an efficient analytical tool for detecting CPF residues in food products. The combination of MWCNT-based signal amplification and the DBAE/[Ru(bpy)_3_]^2+^ quenching-based mechanism enables ultrasensitive performance, rendering the system promising for real-time food safety monitoring and regulatory compliance testing.

## 1. Introduction

Chlorpyrifos (CPF), a commonly used organophosphate pesticide, has raised significant concerns regarding its potential risks to human health and the environment (Rauh et al., 2015). Studies have shown that exposure to CPF during prenatal and early childhood stages can lead to developmental disorders, such as attention deficit hyperactivity disorder, along with increased risks of respiratory illnesses and cancer (Gerunova et al., 2020). The European Food Safety Authority concluded that there is no safe level of exposure to chlorpyrifos, leading to its complete ban in the European Union (European Food Safety Authority, 2019).

Following the EU’s ban on CPF, many countries began to reevaluate their pesticide regulations, leading to the adoption of stricter control measures. However, despite these regulatory efforts, residues of CPF and related substances are still frequently detected in exported fruits and vegetables. This ongoing presence highlights the persistent challenges in effectively enforcing pesticide bans and ensuring compliance across the supply chain (Bakırcı et al., 2014; Deveci et al., 2023; Gormez et al., 2023). Despite these regulatory updates, such pesticides continue to be used in practice.

These challenges have underscored the need for more sensitive and selective detection technologies. Electrochemical sensor systems, particularly electrochemiluminescence (ECL) platforms, offer a portable and highly sensitive approach for detecting pesticide residues (Chen and Wang, 2020). The determination of pesticides using portable sensor systems is of critical importance for environmental and food safety. Portable devices eliminate the need for laboratory analysis, enabling rapid and cost-effective screening, shorter response times, and on-site intervention. The integration of miniaturization, low power consumption, electrochemical and optical transducers, and nanobased and polymer-based selectivity enhancers provides significant improvements in sensitivity and selectivity (Umapathi et al., 2022).

ECL is particularly effective for detecting pesticide residues, including substances like CPF (Wang et al., 2017; Kamyabi and Moharramnezhad, 2020). ECL is widely used for pesticide detection because of its low background, cost-effectiveness, and capability to detect analytes at very low concentrations. A key luminophore in ECL systems is tris(2,2′-bipyridine)ruthenium(II) ([Ru(bpy)_3_]^2+^), which generates highly stable excited-state species during redox reactions. ECL performance can be limited by broad emission peaks, low intensity, and dependence on specific luminophores; these limitations are commonly addressed using coreactants (Forster et al., 2009; Qi et al., 2009). Tripropylamine (TPA) and 2-(dibutylamino)ethanol (DBAE) are commonly used coreactants, with DBAE producing significantly higher ECL signals than TPA (Li et al., 2013).

In electrochemiluminescence systems based on the coreactant mechanism, the analyte can reduce the ECL signal by interfering with the chemical reactions responsible for light emission (Blom et al., 2024). This quenching phenomenon occurs when the analyte either scavenges reactive intermediates, inhibits electron transfer, or absorbs the emitted light (Forster et al., 2009; Blom et al., 2024). Sensor systems leveraging this mechanism are designed to detect analytes by monitoring the decrease in ECL intensity as the analyte concentration increases. Such systems offer distinct advantages, as they enable highly sensitive and specific detection, where even minor changes in analyte levels can significantly alter the ECL signal (Wang et al., 2024). Furthermore, quenching-based ECL sensors exhibit versatility, allowing them to be tailored for diverse applications by selecting the luminophores, coreactants, and electrode materials (Zhao et al., 2019; Wang et al., 2024). This versatility renders them powerful tools for the sensitive detection of low-concentration analytes in complex matrices.

Recent advancements in ECL sensing platforms have enabled ultrasensitive detection of CPF through several innovative approaches. A notable study developed a self-enhanced molecularly imprinted polymer (MIP-ECL) platform based on gold-copper doped Tb-MOFs. This system served as a coreaction promoter, significantly boosting the ECL signal (Fang et al., 2024). Another innovative approach used a ternary nanocomposite consisting of ruthenium nanobeads, silver nanoparticles, and graphene oxide, incorporated onto a glassy carbon electrode. Silver nanoparticles acted as signal enhancers, improving the electrocatalytic efficiency of the electrode (Kamyabi and Moharramnezhad, 2020). [Ru(bpy)_3_]^2+^ species were anchored on the modified electrode surface using chitosan as a crosslinking agent. Additionally, boron nitride quantum dots were introduced as coreactants to further enhance the ECL signal. This electrode showed excellent performance as an ultrasensitive sensor for CPF detection. Both studies highlight the quenching effect of CPF on the ECL signal, whereby increasing CPF concentration lead to a decrease in ECL emission. This quenching effect forms the basis of their sensor designs, in which CPF interacts with the sensor surface, thereby inhibiting the ECL signal.

Functionalizing multiwalled carbon nanotubes (MWCNTs) can significantly enhance the signal intensity in electrochemical systems (Wildgoose et al., 2006). This improvement is largely attributed to the introduction of carboxyl (−COOH) groups, which impart a negative charge to the nanotube surface. These charged functional groups facilitate electron transfer during electrochemical reactions, thereby enhancing overall efficiency (Zhao et al., 2019). Additionally, the presence of −COOH groups increases the hydrophilicity of MWCNTs, improving their dispersion in solution and promoting stronger attachment to the electrode surface. This enhanced adhesion expands the available active area for electrochemical reactions, allowing luminophores such as Ru(bpy)_3_^2+^ and coreactants to interact more effectively. Consequently, these interactions contribute to improved electrochemical performance and a stronger ECL signal (Qi et al., 2009). Additionally, due to their high surface area, MWCNTs provide more active sites, contributing to higher signal intensity (Mao et al., 2010). Functionalized MWCNTs provide more active sites, enhancing ECL reaction efficiency and boosting light emission (Haranath et al., 2012). These features make the sensors more sensitive and accurate, allowing the detection of analytes at much lower concentrations with stronger signals.

Nafion is a polymeric material commonly used in electrochemical sensors due to its excellent properties (Paul Shylendra et al., 2023). It is known for its high ionic conductivity and electroactive surface characteristics. In electrochemical sensors, Nafion enhances the rate of electrochemical reactions on electrode surfaces and increases sensitivity due to its ion-exchange capacity and good electrical conductivity (Haranath et al., 2012). Nafion is typically adsorbed onto the electrode surface, which helps improve sensor stability, allowing for more efficient and precise detection of target analytes (Yoon, 2013; Carolina Torres et al., 2014). Additionally, it is widely used for immobilizing metal ions and adsorbing electrochemically active components, further contributing to sensor performance.

This study presents a quenching-based ECL sensor for CPF. The sensor was designed by immobilizing ruthenium (Ru) complexes and carboxyl-functionalized MWCNTs within a Nafion polymer matrix coated onto a screen-printed carbon electrode (SPCE). This approach improves both the reliability and intensity of the ECL signal, with DBAE serving as a coreactant to further enhance performance. The Ru/MWCNT-COOH/Nafion/SPCE system combined with DBAE as a coreactant is introduced in this study. In the presence of CPF, the ECL reaction between the Ru complexes and DBAE is quenched, reducing in reduced signal intensity (Equations 1–5). By measuring this quenching effect, the sensor can accurately determine CPF concentration. The proposed sensor system was successfully applied for CPF detection in honey samples, demonstrating high sensitivity and accuracy for detecting this hazardous pesticide.

## 2. Materials and methods

### 2.1. Chemicals and instrumentation

All chemicals were of the highest purity available. All chemicals and reagents used in this study, including tris(2,2′-bipyridyl)dichlororuthenium(II) hexahydrate ([Ru(bpy)_3_]Cl_2_·6H_2_O), 2-(dibutylamino)ethanol (DBAE), phosphate-buffered saline (PBS), multiwalled carbon nanotubes (MWCNT), sulfuric acid (H_2_SO_4_), nitric acid (HNO_3_), potassium hexacyanoferrate(III) (K_3_[Fe(CN)_6_]), potassium hexacyanoferrate(II) trihydrate (K_4_[Fe(CN)_6_]·3H_2_O), potassium dihydrogen phosphate (KH_2_PO_4_), and chlorpyrifos, were purchased from Sigma-Aldrich (St. Louis, MO, USA). Nafion (5.0% w/v) solution was obtained from Chemours (Wilmington, DE, USA). Phosphorothioic acid O,O-diethyl O-(3,5,6-trichloro-2-pyridinyl) ester (>98%) was obtained from Cayman Chemical (Ann Arbor, MI, USA).

All electrochemical and ECL analyses were conducted using a portable bipotentiostat/galvanostat combined with an ECL cell (μStat ECL; Metrohm AG, Herisau, Switzerland) equipped with a screen-printed carbon electrode (SPCE) system. The SPCE was modified with an MWCNT-COOH/Ru/Nafion film and served as the working electrode.

A range of laboratory equipment, including a precision balance (ABS220-4 with an accuracy of 0.1 mg; KERN & Sohn GmbH, Balingen, Germany), a pH meter (NeoMet 240-L; ISTEK Inc., Seoul, Republic of Korea), a heated magnetic stirrer (MX-F; Dragon Laboratory Instruments Ltd., Beijing, China), a shaking water bath (WiseBath WSB-30; Memmert GmbH, Schwabach, Germany), an oven (UNB400; Memmert GmbH, Schwabach, Germany), and centrifuges (Centrion Scientific Benchtop K2015R; Centrion Scientific Ltd., Chichester, United Kingdom) and (Beckman Coulter Avanti J-E; Beckman Coulter Inc., Brea, CA, USA), was employed.

### 2.2. Functionalization of MWCNTs

**Figure 1:**
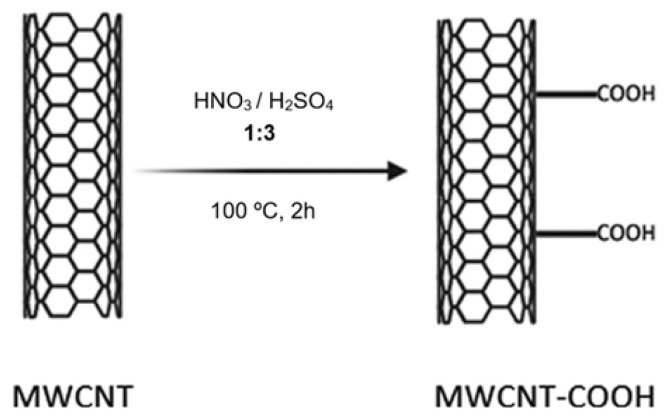
Schematic representation of the functionalization process of multiwalled carbon nanotubes (MWCNTs).

To modify the MWCNTs, 0.4 g of MWCNTs was mixed with a 3:1 H_2_SO_4_/HNO_3_ solution. The mixture was then subjected to sonication in an ultrasonic bath at 100 °C for 2 h. After sonication, the suspension was centrifuged at 7000 rpm for 30 min, and this step was repeated three times. The final suspension was then dried in an oven at 60 °C overnight (Piao et al., 2009; Mallakpour and Soltanian, 2016). This procedure resulted in the preparation of carboxyl-functionalized MWCNTs (MWCNT-COOH) (Figure 1).

### 2.3. Fabrication of the MWCNT-COOH/Ru/Nafion/SPCE

**Figure 2:**
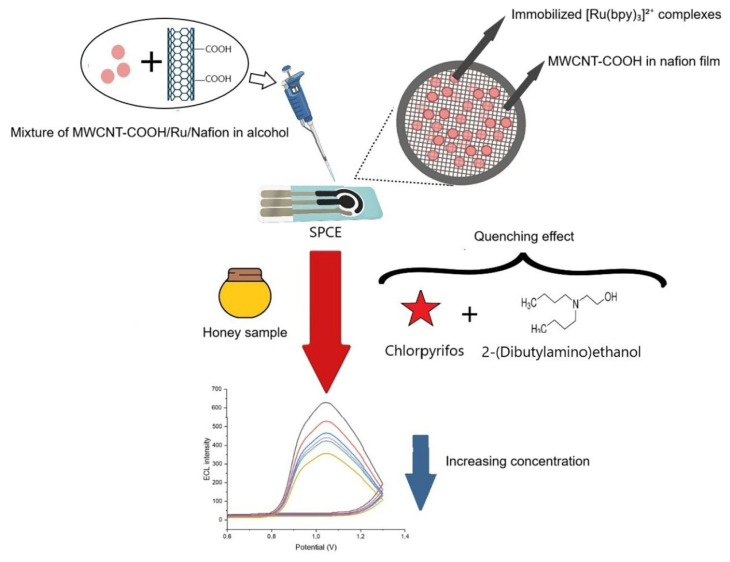
Schematic illustration of the fabrication steps and working mechanism of the MWCNT-COOH/Ru/Nafion-modified SPCE.

To fabricate the modified screen-printed carbon electrode (MWCNT-COOH/Ru/Nafion/SPCE), a suspension of [Ru(bpy)_3_]^2+^ and MWCNT-COOH was prepared in a 0.5% (w/v) Nafion solution, achieving final concentrations of 1 mg/mL [Ru(bpy)_3_]^2+^ and 0.5 mg/mL MWCNT-COOH. A volume of 15 mL of the MWCNT-COOH/Ru/Nafion mixture was carefully applied to the electrode surface, forming a thin film after a 2 h incubation in the dark at room temperature (Figure 2). After modification, the MWCNT-COOH/Ru/Nafion/SPCE system was stored at +4 °C for future analyses.

### 2.4. Electrochemical analysis

The electrochemical behavior of the MWCNT-COOH/Ru/Nafion/SPCE was investigated by cyclic voltammetry (CV) and differential pulse voltammetry (DPV) in the potential range of −0.20 to +0.80 V using 5.0 mM [Fe(CN)_6_]^3−/4−^ in 0.1 M PBS, with scan rates (ν) ranging from 10 to 100 mV s^−1^. The [Fe(CN)_6_]^3−^/^4−^ system exhibits reversible single-electron oxidation behavior. Additionally, analysis of the relationship between oxidation and reduction peak currents across various scan rates facilitated the identification of the optimal scan rate at which the modified SPCE can operate without diffusion limitations (Puthongkham and Venton, 2020). To ensure both rapid analysis and high accuracy, the optimal scan rate and other relevant parameters were determined using CV and DPV methods.

### 2.5. ECL analysis

A coreactant mechanism was employed in the ECL analysis of the designed electrode system. The quenching mechanism follows a pathway similar to that of the TPA coreactant system, owing to their structural resemblance (Shan et al., 2023). During this process, light emission occurs as the luminophore is regenerated; however, the coreactant is consumed during the electrochemical reactions (Equations 1–5).


(1) $$\text{DBAE}\to \text{DBAE}*+e^{-}$$

(2) $$\text{DBAE}{*}^{+}\to \text{DBAE}*+H^{-}$$

(3) $${\text{DBAE}}^{*}+\text{CPF }(\text{quenching effect})\to \text{products of CPF}$$

(4) $$Ru{\left(bpy\right)}_{3}^{2+}-e-\to Ru{\left(bpy\right)}_{3}^{3+}$$

(5) $$Ru{\left(bpy\right)}_{3}^{3+}+\text{DBAE}*\to Ru{\left(bpy\right)}_{3}^{2+}+products$$

(6) $$*Ru{\left(bpy\right)}_{3}^{2+}\to Ru{\left(bpy\right)}_{3}^{2+}+\text{h}\upsilon$$

The [Ru(bpy)_3_]^2+^/DBAE system is utilized in ECL immunoassays, point-of-care testing, and various electrochemical devices (Xue et al., 2009; Yuan et al., 2020). In this setup, [Ru(bpy)_3_]^2+^ was used as the luminophore, while DBAE acted as the coreactant. ECL measurements were performed in 10 mM PBS with a scan rate of 100 mV s^−1^ over a potential range of 0 to 1.3 V. Each measurement was conducted in triplicate, and standard deviations were calculated to ensure data reliability.

### 2.6. ECL detection of CPF residues in honey

ECL analysis measurements were conducted in a honey medium at five different concentrations (1–100 pM) using the MWCNT-COOH/Ru/Nafion/SPE system. To address the viscosity challenges associated with honey samples, the samples were diluted 10 times with 10 mM PBS. The ECL measurements were carried out under optimized conditions, employing a scan rate of 100 mV s^−1^ and a potential range of 0 to 1.3 V. The presence of chlorpyrifos residues reduced the electroactivity of DBAE* (Equation 3), resulting in a decrease in the ECL signal. The sensor operates by establishing a relationship between the increasing concentration of chlorpyrifos residues and the decreasing ECL signal. The ECL signals obtained for chlorpyrifos residues in 10 mM PBS were compared to those measured in honey samples to calculate the relative recovery percentage (% recovery). This value was determined by analyzing the ECL data in the PBS medium and comparing it with the results from the honey samples.

## 3. Results

### 3.1. Functionalization of MWCNTs

**Figure 3:**
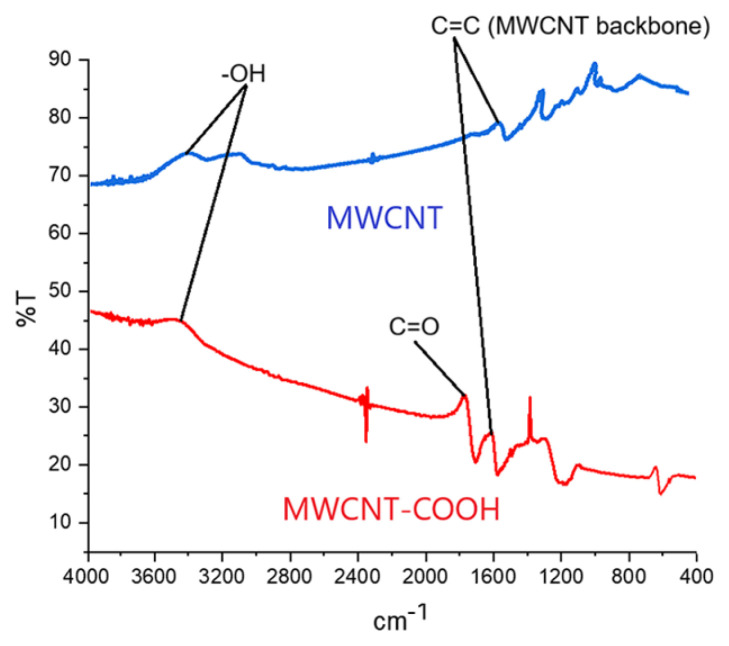
FTIR spectra of pristine MWCNTs and carboxyl-functionalized MWCNTs (MWCNT-COOH).

FTIR analysis was conducted to investigate the structural characteristics of MWCNT-COOH, as shown in Figure 3. Distinct absorption bands were observed at 1632 cm^−1^ and 1700 cm^−1^, corresponding to the stretching vibrations of C=C and C=O bonds, respectively. The prominent peak at 1700 cm^−1^ is indicative of the successful incorporation of carboxyl groups into the MWCNT structure as a result of the modification reaction. These findings indicate that the carboxylation process was successfully achieved.

### 3.2. Scanning electron microscopy analysis

The surface morphology of bare screen-printed electrode (SPE), Nafion-modified SPE, Nafion/MWCNT-COOH–modified SPE, and [Ru(bpy)_3_]^2+^/MWCNT-COOH/Nafion–modified SPE was examined using scanning electron microscopy (SEM).

**Figure 4:**
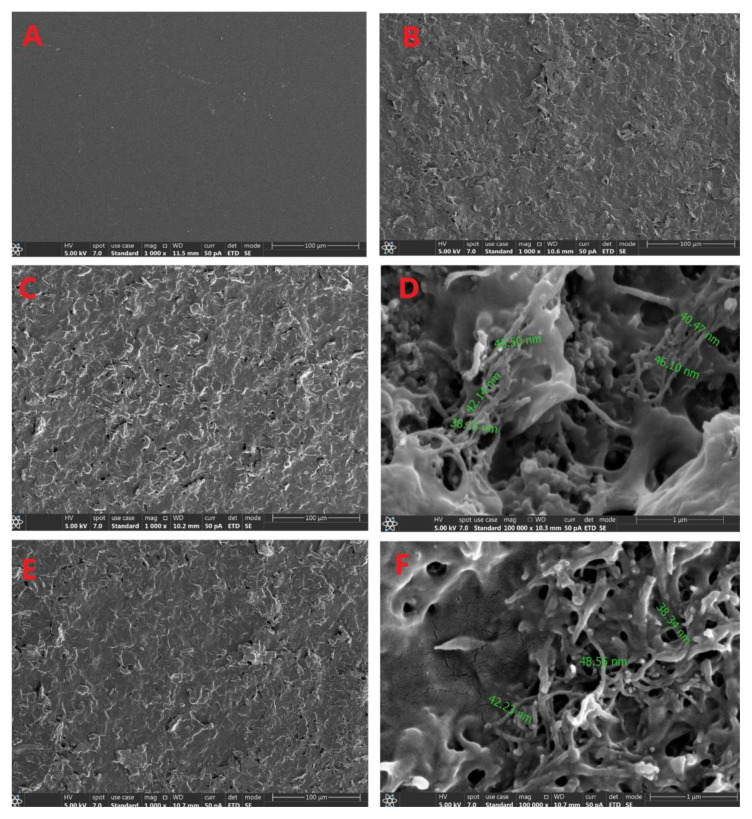
SEM images of (A) bare SPE (1000×), (B) Nafion-modified SPE (1000×), (C) Nafion/MWCNT-COOH–modified SPE (1000×), (D) Nafion/MWCNT-COOH–modified SPE (100,000×), (E) [Ru(bpy)_3_]^2+^/MWCNT-COOH/Nafion–modified SPE (1000×), (F) [Ru(bpy)_3_]^2+^/MWCNT-COOH/Nafion–modified SPE (100,000×).

Scanning electron microscopy (SEM) images were recorded to investigate the surface morphology of the modified electrodes. The images revealed significant changes in the surface morphology after each modification step (Figure 4). These observations indicate that each fabrication step resulted in a uniform and homogeneous modification of the SPE surface. No significant aggregation or irregularities were observed, confirming that the MWCNT-COOH/Nafion/Ru composite was evenly distributed on the electrode. The uniform morphology is expected to contribute to the reproducibility and stability of the electrochemical signal, as well as to the high performance of the sensor system in detecting CPF.

### 3.3. Electrochemical characterization

#### 3.3.1. Determination of the optimum scan rate

**Figure 5:**
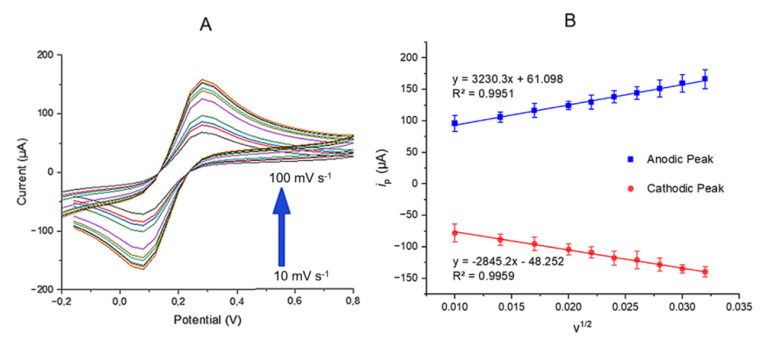
(A) Cyclic voltammograms recorded at scan rates of 10–100 mV s^−1^ for the MWCNT-COOH/Ru/Nafion/SPCE in 5 mM [Fe(CN)6]^3−/4−^ solution. (B) Effect of increasing scan rate (10–100 mV s^−1^) on anodic and cathodic peak currents derived from the CVs.

The electrochemical behavior of the MWCNT-COOH/Ru/Nafion/SPCE was examined (Figure 5A). The [Fe(CN)_6_]^3−^/^4−^ redox system displayed a reversible single-electron transfer process. Moreover, a linear increase in oxidation and reduction peak currents was observed as the scan rate increased. This analysis helped determine the scan rate at which the modified SPCE electrode operates effectively without diffusion limitations. To balance rapid analysis and high accuracy in experimental studies, a scan rate of 100 mV s^−1^ was selected as the optimal value.

In a diffusion-controlled system, the cyclic voltammetry (CV) response is primarily influenced by the potential scan rate, as it dictates the oxidation kinetics of [Fe(CN)_6_]^4−^ to [Fe(CN)_6_]^3−^. Understanding the diffusion coefficient is essential, as it plays a key role in electrochemical behavior. According to the Randles–Sevcik equation, the peak current in diffusion-controlled systems is directly proportional to concentration and increases with the square root of the scan rate (Scholz, 2009; Dokuzparmak et al., 2021). The terms in this equation represent the following parameters: *i*_p_ denotes the peak current, *n* is the number of electrons involved in the redox reaction, *A* represents the electrode surface area, *D_ct_* is the diffusion coefficient of the electroactive species, *C* is the concentration of the species, and ν corresponds to the scan rate (Equation 7). In this equation, evaluating the charge transport rate is critical for comprehending the redox conversion of [Fe(CN)_6_]^4−^ to [Fe(CN)_6_]^3−^ in solution. This rate can be determined through CV measurements by calculating the apparent *D_ct_*. Theoretically, the diffusion coefficient establishes a relationship between molecular diffusion-driven molar flux and the concentration gradient of the electroactive species (Dennany et al., 2011). A higher diffusion coefficient signifies a faster diffusion process between [Fe(CN)_6_]^4−^ and [Fe(CN)_6_]^3−^. Typically, this parameter is derived from an *i_p_* versus *ν*^1^/^2^ plot under diffusion-controlled conditions, which are more pronounced at higher scan rates. In this study, diffusion-controlled behavior was observed between 10 and 100 mV s^−1^, and the corresponding cyclic voltammetric scan rate dependencies, along with Randles–Sevcik plots demonstrating the linear variation of peak current with the square root of the scan rate, are illustrated in Figure 5B.


(7) $$i_{p}=2.65\times 10^{5}n^{3/2}\;AD_{\text{ct}}v^{1/2}C$$

The *D_ct_* values were determined for various scan rates in both cathodic and anodic processes. As depicted in Figure 5B, a clear linear relationship was observed between the peak currents and the square root of the applied scan rate. Notably, the modified SPCE electrode demonstrated a strong linear dependence of oxidation and reduction peak currents on the square root of the scan rate in a 5 mM [Fe(CN)_6_]^3−^/^4−^ solution. This behavior, evident within the 10–100 mV s^−1^ range, confirms the diffusion-controlled nature of the electrochemical process.

### 3.4. Effect of surface modifications on sensor performance

**Figure 6:**
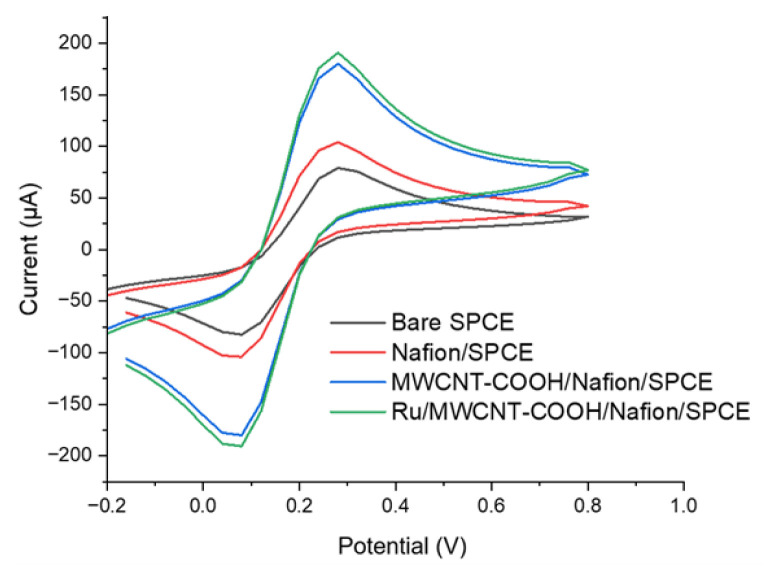
Cyclic voltammograms of bare SPCE, Nafion/SPCE, MWCNT-COOH/Nafion-SPCE, and MWCNT-COOH/Ru/Nafion/SPCE recorded in 5 mM [Fe(CN)_6_]^3−/4−^ solution in 0.1 M PBS.

The effect of Nafion concentration, MWCNT-COOH concentration, and [Ru(bpy)_3_]^2+^ concentration at their optimal values were examined by CV measurements for each surface modification. As depicted in Figure 6, the bare SPCE electrode showed an increase in current upon the addition of 0.5% (w/v) Nafion, which can be attributed to the conductive properties of the Nafion polymer (Wang, 2002; Bertoncello et al., 2007; Dokuzparmak et al., 2021). The introduction of 0.5 mg/mL MWCNT-COOH onto the electrode surface also resulted in a rise in current, a change expected due to the combination of enhanced surface area and the conductive characteristics of MWCNT-COOH (Niu et al., 2007; Wulandari et al., 2018). Following the modification of the electrode surface with [Ru(bpy)_3_]^2+^ complexes, no meaningful electrochemical change was observed. However, the modified electrode surface with [Ru(bpy)_3_]^2+^ was further studied through ECL experiments.

### 3.5. Effect of Nafion concentration

Nafion polymers are extensively utilized in electrochemical applications due to their exceptional chemical resistance, especially in harsh environments containing strong oxidizing agents. As a widely used cation-exchange polymer, Nafion is favored for its ability to selectively interact with large hydrophobic cations, a property attributed to its sulfonic acid groups, which facilitate the incorporation of electroactive cations from the solution phase into the polymer matrix (Mauritz and Moore, 2004; Cele and Ray, 2009). This selectivity plays a crucial role in various electrochemical and sensing applications, where controlled ion transport and stability are essential. However, despite its beneficial characteristics, excessive Nafion concentrations on electrode surfaces can lead to diffusion-related limitations. While Nafion exhibits intrinsic conductivity due to its ionic nature, an overly thick Nafion layer can act as a diffusion barrier, hindering mass transport of electroactive species to the electrode surface. This can result in reduced current responses in sensor systems at higher Nafion concentrations (Jung et al., 2020). To mitigate this issue, it is essential to optimize Nafion concentration in sensor designs to achieve a balance between conductivity and efficient mass transport.

**Figure 7:**
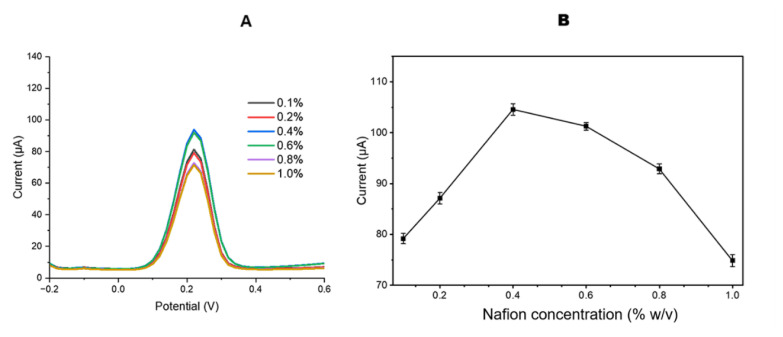
(A) DPV measurements and (B) corresponding peak currents of 5 mM [Fe(CN)_6_]^3−^/^4−^ obtained using Nafion concentrations ranging from 0.1% to 1% (w/v) recorded over a potential range of −0.20 to +0.60 V at a scan rate of 100 mV s^−1^.

A Nafion film solution was prepared using Nafion dissolved in methanol at concentrations ranging from 0.1% to 1% (w/v). Subsequently, 15 μL of these methanol-based solutions were drop-cast onto the surface of each SPCE. The coated electrodes were left to dry at room temperature for 2 h before being stored at +4 °C for future analysis. Differential pulse voltammetry (DPV) measurements were conducted. The highest signal response was recorded at a Nafion concentration of 0.4% (w/v) (Figure 7), and the optimal Nafion concentration was determined to be 0.4% (w/v) based on Figure 7B. Beyond this concentration, a decline in current signals was observed, likely due to diffusion limitations affecting [Fe(CN)_6_]^3−^/^4−^ ion transport. These findings align well with previously reported literature (Wang, 2002; Bertoncello et al., 2007; Dennany et al., 2011; Nguyen et al., 2020).

### 3.6. Effect of Nafion film thickness

**Figure 8:**
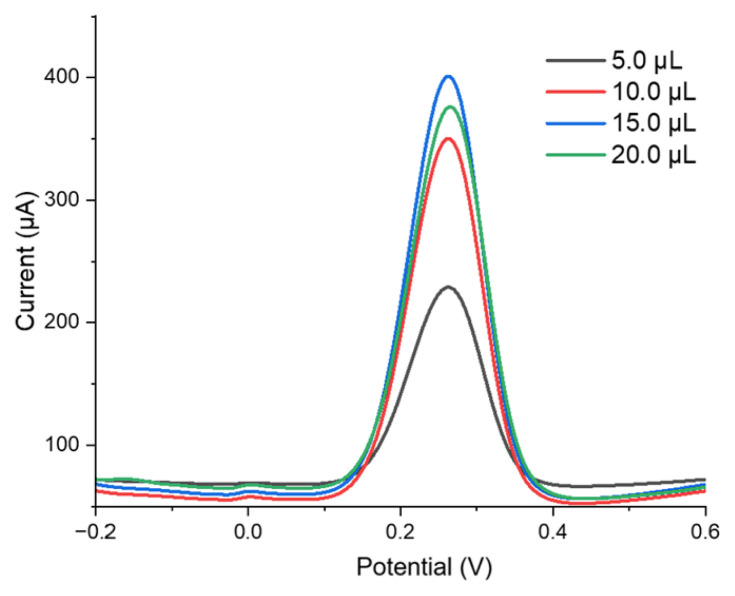
The impact of different volumes (5–20 μL) of Nafion on DPV responses in a 5 mM [Fe(CN)_6_]^3−^/^4−^ solution.

The thickness of the Nafion film plays a crucial role in determining both the peak current and the repeatability of measurements (Adhikari et al., 2021; Chadha et al., 2021). To optimize the performance of the MWCNT-COOH/Ru/Nafion/SPCE system, the ideal film thickness was systematically investigated. Nafion solutions with a concentration of 0.4% (w/v) were applied to the SPCE surface in varying volumes of 5, 10, 15, and 20 μL. As shown in Figure 8, the highest current response was obtained when 15 μL of Nafion solution was applied. However, at 20 μL, a decline in current was observed, likely due to diffusion limitations caused by excessive film thickness, which hindered [Fe(CN)_6_]^3−^/^4−^ ion transport and slowed down the reaction. Based on these findings, 15 μL was determined to be the optimal Nafion volume for further studies.

Previous studies in the literature have reported similar trends. For instance, a Nafion/platinum-modified electrode was developed for trace iron detection, where Pt electrodes coated with Nafion films of varying thickness underwent electrochemical analysis in an electrolyte containing 100 ppb Fe(III) (Nguyen et al., 2020). Atomic force microscopy measurements revealed that as the Nafion thickness increased from 70 to 210 nm, the signal response improved. However, despite achieving the highest current, the 210 nm film exhibited poor repeatability, whereas the 90 nm film not only provided a strong signal but also ensured excellent measurement consistency (Nguyen et al., 2020). Our findings align with these observations, demonstrating an increase in DPV response up to 15 μL of 0.4% Nafion solution, followed by a decline at 20 μL due to diffusion restrictions (Figure 8).

### 3.7. ECL characterization

#### 3.7.1. Effect of [Ru(bpy)_3_]^2+^ concentration

In the modified optical sensor system designed within the scope of the project, [Ru(bpy)_3_]^2+^ was used as the luminophore. Ruthenium complexes commonly used in ECL systems are known for their high emission efficiency and excellent luminescence properties (Dokuzparmak et al., 2021). These superior characteristics make them especially important in electrochemiluminescence (ECL) applications. The concentration of the ruthenium complex immobilized in the Nafion film is a critical parameter for the performance of the ECL system and must be optimized.

**Figure 9:**
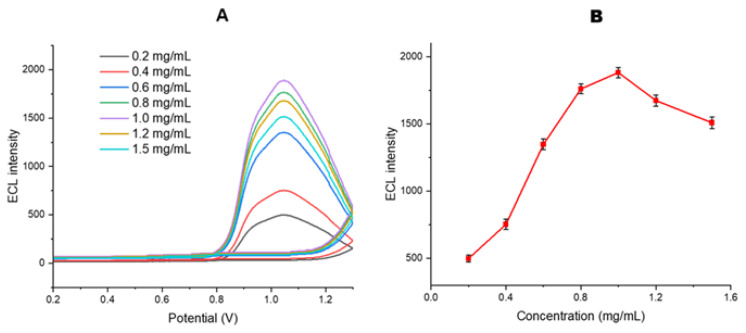
Effect of varying [Ru(bpy)_3_]^2+^ concentrations (0.1–1.5 mg mL^−1^) on the MWCNT-COOH/Nafion/Ru/SPCE system: (A) ECL signal measurements and B) comparison of ECL peak intensities recorded in the presence of 40 mM DBAE in 10 mM PBS (pH 7.0).

The effect of [Ru(bpy)_3_]^2+^ concentration on the ECL signal intensity was studied as part of the optimization of the MWCNT-COOH/Nafion/Ru/SPCE sensor surface. The surfaces of MWCNT-COOH/Nafion/Ru/SPCE were prepared with varying concentrations of [Ru(bpy)_3_]^2+^ ranging from 0.1 to 1.5 mg/mL, and ECL analyses were performed in the presence of 40 mM DBAE as the coreactant in 10 mM PBS at pH 7.0 (Figure 9A).

At the optimal scan rate of 100 mV s^−1^ and within the potential range of 0 to 1.3 V, significant increases in ECL signal were observed as the concentration of [Ru(bpy)_3_]^2+^increased from 0.1 to 1.0 mg/mL (Figure 9B). However, at concentrations of 1.0 and 1.5 mg/mL [Ru(bpy)_3_]^2+^, a slight decrease in the ECL signal was noted. This reduction in the ECL signal at higher concentrations can be attributed to the aggregation of the luminophore complexes or the occurrence of self-quenching, where excited molecules undergo quenching due to charge–charge interactions (Takahashi and Jin, 2008; Huang et al, 2013).

When evaluating the ECL results, it was observed that the [Ru(bpy)_3_]^2+^ complexes in the MWCNT-COOH/Nafion/Ru/SPCE system are capable of rapid electron transfer and can generate high ECL signals. As a result, the [Ru(bpy)_3_]^2+^ complexes were optimized to produce the highest possible ECL signal in aqueous solutions. The optimal concentration of [Ru(bpy)_3_]^2+^ complex required to achieve the highest ECL signal was determined to be 1.0 mg/mL.

#### 3.7.2. Effect of coreactant concentration on the ECL signal

Higher ECL signals can be obtained in the presence of different coreactants. The use of a coreactant that produces higher ECL signals can lead to lower limits of detection (LoD) and quantification (LoQ) for the designed modified SPCE system. As an alternative to TPA, the most commonly used coreactant in the literature, DBAE can be employed (Huang et al., 2013). Considering that DBAE is an aliphatic tertiary amine, the quenching mechanism proposed for the [Ru(bpy)_3_]^2+^/TPA system could also be applicable to the [Ru(bpy)_3_]^2+^/DBAE ECL system (Xue et al., 2009; Yuan et al., 2020). In this process, DBAE first forms a DBAE* cationic radical through oxidation at the electrode, which subsequently transforms into a DBAE* free radical. Due to the strong reducing ability of DBAE* free radicals, the ruthenium complex is reduced, generating excited Ru* species, which subsequently return to the [Ru(bpy)_3_]^2+^ ground state through light emission (Huang et al., 2013). Chlorpyrifos reduces the luminophore optical signal by interfering with radical generation from the coreactant, likely due to steric hindrance effects (Jiao et al., 2017). Within this mechanism, electron transfer between DBAE* and CPF inhibits electron transfer between active DBAE* radicals and Ru* species. Consequently, the ECL quenching effect increases with increasing CPF concentration, resulting in a progressive decrease in the ECL signal (Equations 1–5).

**Figure 10:**
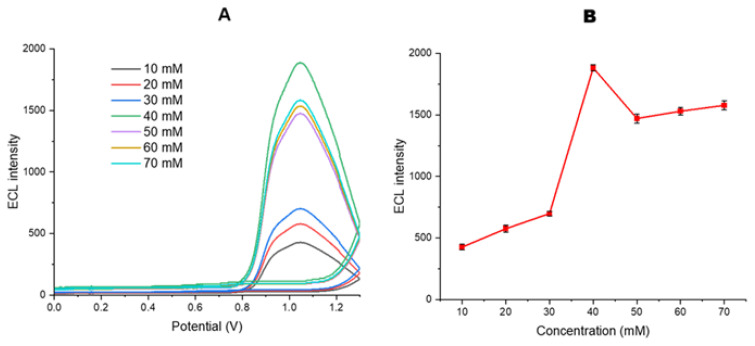
(A) ECL signals recorded in the presence of DBAE at different concentrations and (B) comparison of ECL peak intensities, measured over a potential range of 0–1.3 V at a scan rate of 100 mV s^−1^ in 10 mM PBS (pH 7.0).

The MWCNT-COOH/Nafion/Ru/SPCE electrode (containing 0.75 mg/mL MWCNT, 0.5% Nafion (w/v), and 1 mg/mL [Ru(bpy)_3_]^2+^) was prepared, and ECL intensities were examined in the presence of different DBAE concentrations (10, 20, 30, 40, 50, 60, and 70 mM) in 10 mM PBS (pH 7.0) (Figure 10A). The optimal coreactant concentration for the designed sensor system was determined to be 40 mM (Figure 10B).

Upon reviewing similar studies in the literature, DBAE has a high electron transfer capability and can stably produce high ECL signals in aqueous solutions (Liu et al., 2007; Kirschbaum-Harriman et al., 2017). Additionally, ECL intensity studies demonstrated that DBAE can generate high electrical currents and produce a strong ECL signal (Figure 10). The highest ECL signal was observed at 40 mM DBAE; therefore, this concentration was selected as the optimum coreactant to achieve maximum signal intensity and low LoD and LoQ values for the sensor system. At concentrations higher than 40 mM, a slight decrease in the ECL signal was observed, which is likely attributable to the self-quenching effect of DBAE. Quenching generally occurs through energy or electron transfer. The quenching effect, known as Förster resonance energy transfer, occurs over short distances (typically 1–10 nm) through direct electrodynamic interactions between reactants (Parveen et al., 2013). In other words, when coreactants are at higher concentrations and are excited, they are in closer proximity to each other, allowing energy transfer and resulting in quenching. Consequently, electron transfer occurs less efficiently between the excited coreactant and [Ru(bpy)_3_]^2+^, leading to a decrease in the ECL signal (Parveen et al., 2013). In conclusion, at concentrations higher than 40 mM, DBAE undergoes energy transfer between coreactant molecules via Förster resonance energy transfer, leading to partial quenching and a decrease in the ECL response (Figure 10).

#### 3.7.3. Effect of MWCNT-COOH concentration on the ECL signal

**Figure 11:**
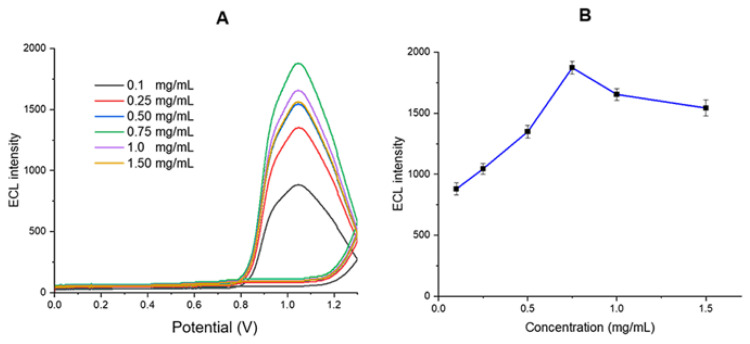
(A) ECL measurement results and (B) corresponding ECL peak intensities for different MWCNT-COOH concentrations (0.0–0.75 mg/mL) recorded in the presence of 40 mM DBAE in 10 mM PBS (pH 7.0).

It has been reported that the use of carbon nanotubes (CNTs or MWCNTs) increases the ECL signal intensity of ruthenium-based luminophores (Li et al., 2012). Additionally, the conductivity of MWCNTs can be enhanced through functionalization (Wulandari et al., 2018). Modification with MWCNTs leads to an increased electrode surface area and enhanced electron transfer between [Ru(bpy)_3_]^2+^ and DBAE, resulting in an increase in ECL signal intensity. The change in ECL signal intensity of the modified SPCE electrode system, containing 1.0 mg/mL [Ru(bpy)_3_]^2+^, a 0.5% (w/v) Nafion film, and varying concentrations of MWCNT-COOH (0.1–1 mg/mL), was investigated by ECL analysis in the presence of 40 mM DBAE (Figure 11).

The ECL signal intensity of the system in the absence of CNTs (0 mg/mL) was slightly lower. However, at a CNT concentration of 0.75 mg/mL, the ECL signal intensity is observed to be at its highest level. At higher CNT concentrations (1.0 and 1.5 mg/mL), the ECL signal intensity decreases slightly. This decrease may be attributed to the increased amount of carbon nanotubes (CNTs) absorbing and scattering the ECL emission within the Nafion film, leading to a reduction in the detected ECL signal (Li et al., 2012). The optimum CNT concentration for achieving the highest ECL signal was determined to be 0.75 mg/mL.

#### 3.7.4. Effect of pH on the ECL signal

**Figure 12:**
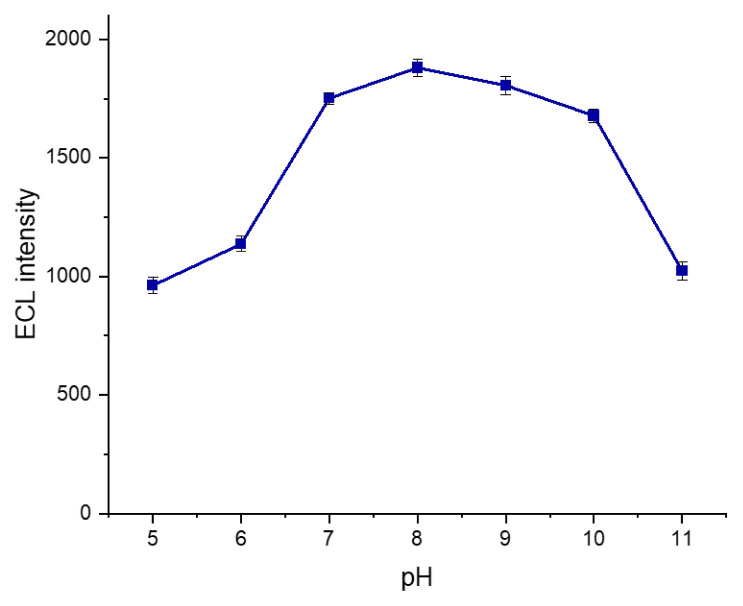
Effect of pH on the ECL intensity of the MWCNT-COOH/Nafion/Ru/SPCE system measured in 0.1 M PBS containing 40 mM DBAE over a pH range of 5.0–11.0, within a potential range of 0–1.3 V at a scan rate of 100 mV s^−1^.

In ECL-based systems, pH is a crucial factor. The pH of the medium directly influences the rate of reactions occurring (Li et al., 2013). To obtain the highest ECL signal from the MWCNT-COOH/Nafion/Ru/SPCE system, ECL measurements were conducted under optimized conditions across different pH environments. To investigate the effect of varying pH on the modified electrode system, experiments were performed in PBS solutions within the pH range of 5.0 to 11.0. The resulting ECL data are shown in Figure 12.

As shown in Figure 12, the modified electrode system exhibited enhanced ECL signals in environments with pH values between 7.0 and 10.0. A partial decrease in ECL signal intensity was observed at pH 11.0. This decrease may be attributed to the requirement for DBAE deprotonation prior to its reaction with ruthenium complexes. Proton removal from DBAE facilitates more efficient redox reactions with the ruthenium complex (Yuan et al., 2020). In summary, at higher pH values, partial dissociation of reactive species may reduce the availability of ECL-active reactants, thereby decreasing the ECL intensity. Notably, the highest ECL signal intensity was observed in the pH 8.0 PBS environment. The peak at pH 8.0 can be explained by the facilitated deprotonation of DBAE, which enables more efficient redox interactions with ruthenium complexes.

### 3.8. Analytical performance of the modified sensor system

**Figure 13:**
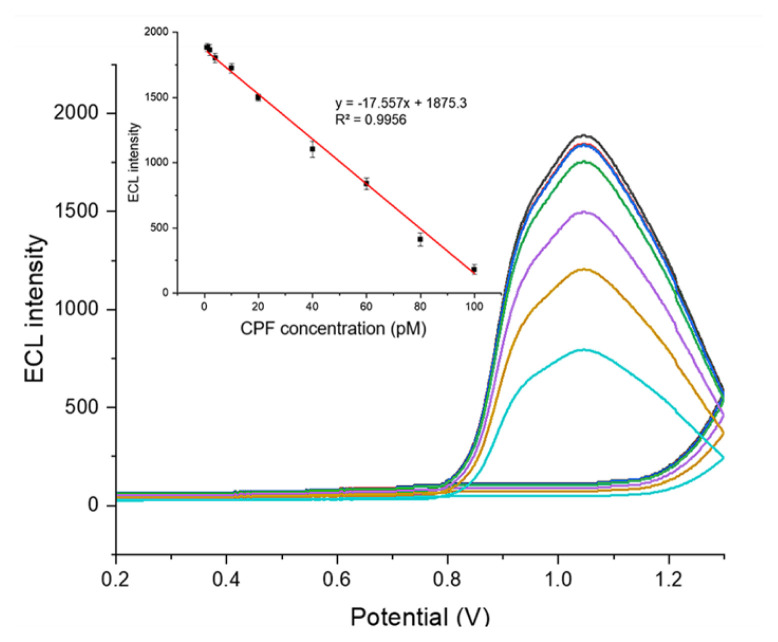
ECL signal intensities obtained in the presence of 40 mM DBAE for CPF concentrations ranging from 1 to 100 pM in 10 mM PBS (pH 8.0), recorded over a potential range of 0–1.3 V at a scan rate of 100 mV s^−1^.

To evaluate the analytical performance of the designed MWCNT-COOH/Nafion/Ru/SPCE sensor system, CPF solutions at concentrations ranging from 1 pM to 100 pM were prepared in the presence of 40 mM DBAE in 10 mM PBS (pH 8.0). ECL measurements were taken, and the relationship between CPF concentration and the corresponding ECL signal intensity was analyzed (Figure 13).

Based on the ECL signal intensities obtained at varying CPF concentrations, the MWCNT-COOH/Nafion/Ru/SPCE system exhibited a limit of detection (LoD) of 0.24 ± 0.05 pM (2.4 × 10^−13^ M; 3.3 × SD) and a limit of quantification (LoQ) of 0.73 ± 0.13 pM (7.3 × 10^−13^ M; 10 × SD). A linear working range with a constant slope was observed between 0.73 ± 0.13 pM and 100 ± 1.49 pM, with a correlation coefficient of R^2^ = 0.9956 (Figure 13). The sensor system demonstrated high sensitivity for CPF detection, indicating its suitability for precise determination of CPF over a wide concentration range with high selectivity and accuracy.

The MWCNT-COOH/Nafion/Ru/SPCE system offers lower LoD and LoQ values compared to many similar studies reported in the literature (Table 1), indicating its high sensitivity. Additionally, the system demonstrates a linear working range over a wide concentration range, highlighting its strong potential for real-world sample analysis. The system is particularly suitable for detecting CPF at pM concentration levels and can be effectively applied to real sample analyses.

### 3.9. Selectivity studies

Selectivity of the designed sensor system was systematically evaluated by performing ECL measurements in the presence of several commonly used pesticides alongside CPF (Kamyabi and Moharramnezhad, 2021). Experiments were conducted under optimized conditions, with a concentration of 40 pM applied for both CPF and each potential interfering species. The standard addition method was employed using acetamiprid (AM), deltamethrin (DLM), fenpropathrin (FPP), and fenvalerate (FENV) to assess the sensor’s selectivity.

**Figure 14:**
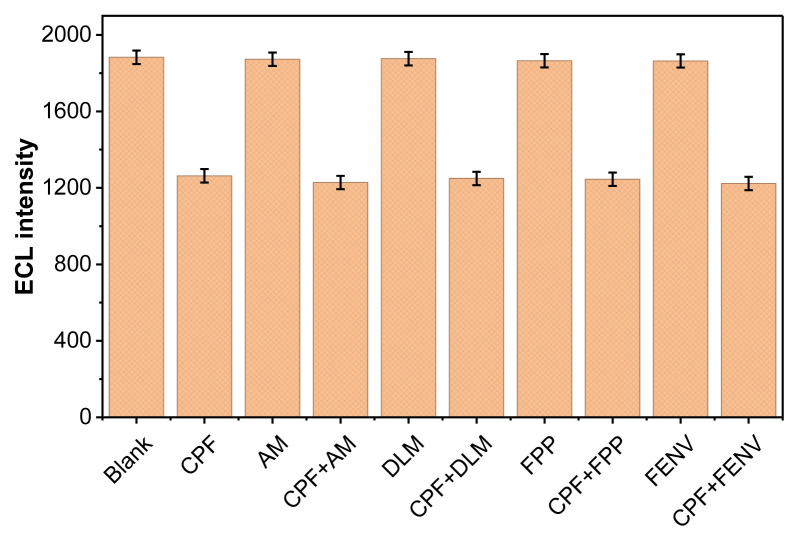
ECL intensity of the modified electrode measured in 0.1 M PBS (pH 8.0) containing 40 mM DBAE in the absence and presence of 40 pM CPF and potential interfering pesticides, including acetamiprid (AM), deltamethrin (DLM), fenpropathrin (FPP), and fenvalerate (FENV).

As shown in Figure 14, no noticeable variation in ECL intensity was observed upon the addition of the interferents together with CPF, indicating that the developed sensor exhibits high selectivity toward CPF detection under the optimized conditions. When compared with similar systems reported in the literature (Kamyabi and Moharramnezhad, 2021; He et al., 2022), the obtained results indicate that the proposed sensor exhibits high selectivity. This finding also suggests that the presence of the coreactant DBAE contributes to the observed selectivity toward CPF determination.

### 3.10. Repeatability and reproducibility studies

**Figure 15:**
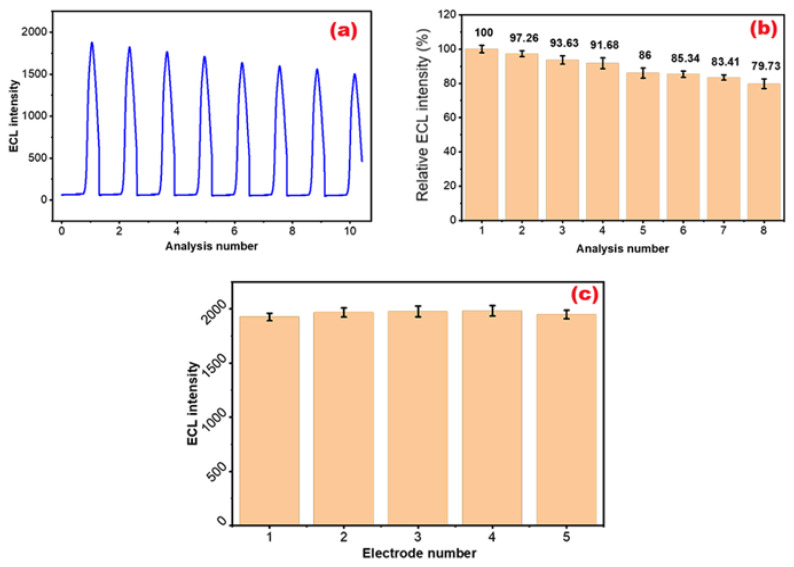
(A) ECL intensity, (B) relative ECL intensity (%), and (C) electrode-to-electrode reproducibility of the MWCNT-COOH/Nafion/Ru/SPCE system evaluated using five independently prepared electrodes (n = 5) in the presence of 40 mM DBAE and 40 pM CPF in 10 mM PBS (pH 8.0), measured over a potential range of 0–1.3 V at a scan rate of 100 mV s^−1^.

The repeatability of the designed sensor system was evaluated through consecutive measurements under optimized conditions. A 30 min interval was maintained between each measurement (De Assis et al., 2025). A total of eight consecutive measurements (n = 8) were performed on the same electrode at a representative CPF concentration. The ECL signals were used to calculate the mean and standard deviation (SD), indicating good repeatability of the sensor response (Figures 15A and 15B). The initial ECL signal was compared with signals obtained in subsequent measurements, and the point at which the signal decreased to 80% of its initial value was considered the reference threshold for repeatability assessment (Figure 15). Accordingly, no further measurements were performed once the signal decreased to 80% of its initial value, since values below this level were regarded as unreliable for detection.

Additionally, reproducibility of the sensor system was evaluated using five independently prepared electrodes (n = 5) measured on the same day under identical experimental conditions (Figure 15C). The obtained ECL signals were used to calculate the relative standard deviation (%RSD). %RSD of the sensor was determined to be approximately 1.8%, demonstrating the high reproducibility of the sensor fabrication process and the consistent performance of independently prepared electrodes.

### 3.11. Storage stability studies

The long-term storage stability of the sensor system was evaluated by performing ECL measurements on independently but identically fabricated electrodes (n = 5) at each storage time point (1, 3, 7, 10, 15, and 30 days). The electrodes were stored at room temperature and in the dark to avoid environmental and photochemical degradation. For each storage day, the ECL intensity and relative standard deviation (%RSD) were calculated to assess both interelectrode variation and signal stability.

It was observed that the %RSD increased only slightly from 1.81% on day 1 to 2.90% on day 30, indicating minimal variation between electrodes and high stability of the ECL signal throughout the storage period (Table 2). These results demonstrate that the sensor system maintains stable performance during long-term storage, highlighting its potential suitability for practical field applications.

### 3.12. Analysis of honey samples

**Figure 16:**
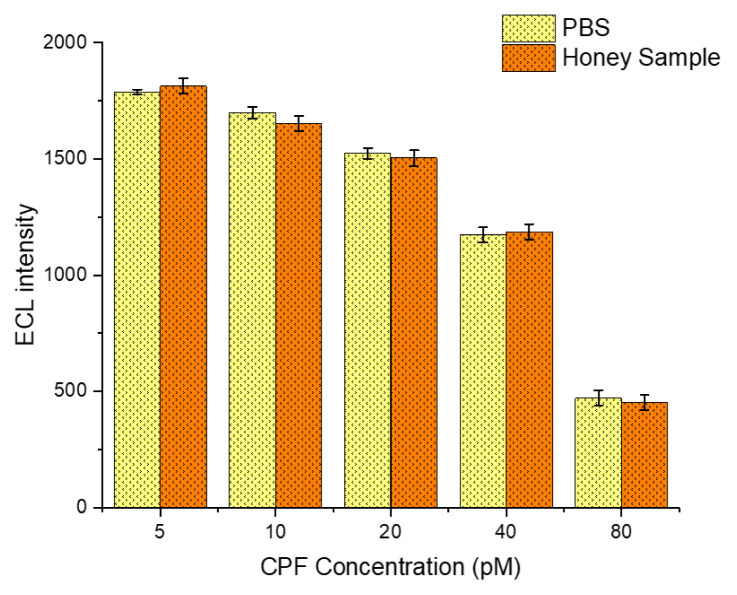
Percentage recovery results of ECL signals obtained from honey samples spiked with CPF at various concentrations (5, 10, 20, 40, and 80 pM), measured over a potential range of 0–1.3 V at a scan rate of 100 mV s^−1^.

Recent studies have shown that CPF can be found in the roots, leaves, and fruits of many agricultural plants (Nandhini et al., 2021). Bees collect pollen and nectar containing CPF from these plants and carry it back to the hive, leading to CPF contamination in honey (Villalba et al., 2020). Therefore, an experimental setup has been designed to detect CPF in honey samples using the MWCNT-COOH/Nafion/Ru/SPCE system, enabling the determination of CPF levels in real honey samples. The MWCNT-COOH/Nafion/Ru/SPCE system was applied for the analysis of honey samples. To mitigate viscosity-related issues, honey samples were diluted 10-fold with 10 mM PBS before performing ECL measurements. CPF was spiked into the honey samples using the standard addition method. ECL analyses were conducted under optimized conditions for honey samples containing five different CPF concentrations (5, 10, 20, 40, and 80 pM). The ECL signal intensities obtained from honey samples were compared with those acquired in PBS, and % recovery values were calculated (Figure 16).

The analysis demonstrated that CPF concentrations in honey samples closely matched those measured in PBS during ECL assessments (Figure 13). The percentage recovery varied between 96.26 ± 3.78% and 101.45 ± 4.16%. These results indicate that the MWCNT-COOH/Nafion/Ru/SPCE system is accurate and sensitive for detecting CPF in honey, underscoring its potential for practical analytical applications.

## 4. Discussion

The study introduces a novel approach for chlorpyrifos detection using an ECL sensor system based on carboxyl-modified MWCNTs and [Ru(bpy)_3_]^2+^ immobilized on the SPCE surface with a Nafion polymer. Nafion polymer plays a crucial role in both enhancing the electrochemical signal intensity and facilitating the immobilization of carboxyl-modified MWCNTs and ruthenium complexes. Its unique properties help achieve a more stable dispersion of the carboxyl-modified MWCNTs, creating an ideal environment for the immobilization of ruthenium complexes, which in turn leads to higher ECL signal intensities.

A key aspect of this study is the efficient use of the [Ru(bpy)_3_]^2+^/DBAE coreactant mechanism to generate a quenching effect for CPF detection, which is applied for the first time in this context. This approach enhances the portability and practicality of the sensor, as it does not require complex instrumentation or multistep sample preparation. The combination of carboxyl-modified MWCNTs and [Ru(bpy)_3_]^2+^ within the Nafion matrix further enhances dispersion and results in more intense ECL signals, making the system effective for CPF detection.

In terms of reproducibility and stability, the sensor demonstrated excellent intraelectrode repeatability with consecutive measurements showing minimal variation (RSD < 2%). Additionally, interelectrode reproducibility across five independently prepared electrodes resulted in a %RSD of 1.8%, indicating consistent fabrication and performance. Storage stability studies further confirmed that the sensor maintains signal integrity over a 30 day period, with %RSD values increasing only minimally from 1.8% to 2.9%, reflecting high robustness and suitability for field deployment.

Selectivity tests also confirmed the sensor’s high specificity toward CPF, with negligible interference from other common pesticides and sample matrix components. This combination of high selectivity, excellent reproducibility, and robust stability underscores the sensor’s suitability as a simple, portable, and reliable platform for real-world pesticide detection.

Analytically, the sensor exhibited a very low limit of detection (LoD) of 0.24 ± 0.05 pM and a wide linear dynamic range suitable for practical applications. The successful application in honey samples demonstrated consistent recovery rates, emphasizing the system’s accuracy and applicability in real food matrices. Overall, the study highlights the practical advantages of this ECL sensor, combining simplicity, portability, high performance, and strong analytical reliability, and represents a significant advancement in CPF monitoring for food safety.

## Figures and Tables

**Table 1:** Analytical performance of previously reported methods for CPF determination. (PEC: photoelectrochemical; GCE: glassy carbon electrode; MIP-ECL: molecularly imprinted polymer-based electrochemiluminescence; FL: fluorescence; CV: cyclic voltammetry).

Electrode	Method	LoD (Molar)	Linear dynamic range (molar)	Reference
MWCNT-COOH/Nafion/[Ru(bpy)_3_]^2+^/SPCE	ECL	2.4 × 10^−13^	7.3 × 10^−13^ to 1 × 10^−10^	Present study
Pt–Au/MWCNT-modified GCE	ECL	3.0 × 10^−8^	5.0 × 10^−8^ to 5 × 10^−7^	(Miao et al., 2016)
PFBT-PNPs-modified GCE	ECL	1.5 × 10^−13^	1.0 × 10^−12^ to 1 × 10^−7^	(He et al., 2020)
Au@Cu:Tb-based MOFs	MIP-ECL	0.083 × 10^−12^	0.28 × 10^−12^ to 10^−6^	(Fang et al., 2024)
P3HT–TiO_2_-modified GCE	PEC	1.0 × 10^−8^	2.0 × 10^−7^ to 1.6 × 10^−6^	(Li et al., 2011)
Au–PPy–rGO-modified GCE	Amperometry	5.0 × 10^−11^	1.0 × 10^−9^ to 5 × 10^−6^	(Yang et al., 2014)
Upconversion nanoparticle-based FL assay	FL	1.91 × 10^−11^	5.7 × 10^−11^ to 5.7 × 10^−9^	Lin et al., 2021)
Apt-functionalized GO@Fe_3_O_4−_CB modified GCE	CV	9.41 × 10^−12^	2.85 × 10^−11^ to 0.28	(Jiao et al., 2017)

**Table 2:** Storage stability of the MWCNT-COOH/Nafion/Ru/SPCE sensor system evaluated using five independently prepared electrodes (n = 5) stored at room temperature in the dark for 1, 3, 7, 10, 15, and 30 days. ECL measurements were conducted in the presence of 40 mM DBAE and 40 pM CPF in 10 mM PBS (pH 8.0), over a potential range of 0–1.3 V at a scan rate of 100 mV s^−1^).

Day	Mean (ECL intensity (a.u.))	SD	%RSD
1	2015	36.48	1.81
3	1975	39.13	1.98
7	1981	44.19	2.23
10	1964	46.36	2.36
15	1941	51.66	2.66
30	1955	56.78	2.90
